# Optical Coherence Tomography Velocimetry for In-Line Processing: Velocity Profiles and the Intermittency of Opaque Complex Fluids In Situ

**DOI:** 10.1021/acsengineeringau.4c00043

**Published:** 2025-02-10

**Authors:** Owen Watts Moore, Thomas Andrew Waigh, Ali Arafeh, Philip Martin, Cesar Mendoza, Adam Kowalski

**Affiliations:** † Biological Physics, Department of Physics and Astronomy, The University of Manchester, Manchester M13 9PL, U.K.; ‡ Department of Chemical Engineering, The University of Manchester, Manchester M13 9PL, U.K.; § Unilever Research & Development, Port Sunlight Laboratory, Quarry Road East, Bebington, Wirral CH63 3JW, U.K.; ∥ Photon Science Institute, The University of Manchester, Manchester M13 9PL, U.K.

**Keywords:** optical coherence tomography velocimetry, in-line processing, lamellar gel network, velocity profile, turbulence, power law fluid, rheology

## Abstract

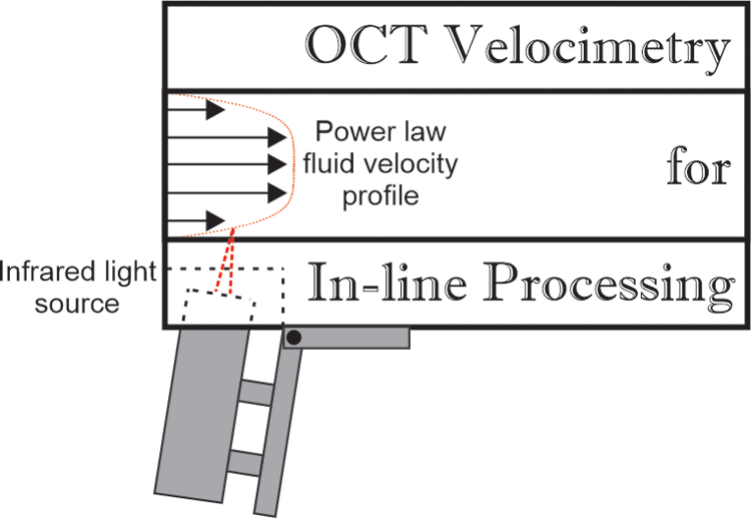

We demonstrate optical coherence tomography (OCT) velocimetry with in-line processing of complex fluids for the first time. The OCT measurements were performed on a perspex section of a test rig containing ∼40 L of complex fluids, analogous to real-world manufacturing conditions. Opaque solutions of lamellar surfactant gel networks (LGNs) and powdered milk were explored. Velocity profiles characteristic of power law fluids were found in the LGNs, in good agreement with independent measurements of the flow rate and off-line determination of viscosity. The velocity fluctuations of 3.4 pL volumes of the fluids in the test rig were also explored. LGNs demonstrated smooth, steady flows, whereas the powdered milk demonstrated marked instability, both showing intermittent behavior and Kolmogorov scaling for fully developed classical turbulence of Newtonian fluids (*P*(ω) ∼ ω^–5/3^, where *P*(ω) is the power spectral density of the velocity fluctuations, and ω is the frequency). The effects of dynamic changes in formulation on velocimetry measurements could be observed with LGNs during the addition of salt and with the milk powder due to biofouling.

## Introduction

Many industrially relevant complex fluids and soft solids are formulated in pipelines during their manufacturing, e.g., conditioners, shampoos, foods, paints, antibodies, vaccines, and so forth. Monitoring the behavior of these materials dynamically and in situ promises large improvements in efficiency, wastage, quality control, and costs. For quality control measurements based on flow behavior (rheology), samples typically need to be examined ex situ. Off-line rheometer measurements are time-consuming (making the dynamic formulation of fluids more challenging), and they may not be representative of the complex fluids during production due to aging or geometrical effects. Many high-resolution, noninvasive methods to monitor complex fluids are based on photonics, e.g., infrared spectroscopy, Raman spectroscopy, UV spectroscopy, or laser Doppler velocimetry. However, all of these techniques are affected by the multiple scattering of light that inevitably occurs with opaque fluids. It is hard to quantify physical parameters (e.g., flow velocity or concentration) based on these methods in the multiple-scattering regime. A recent advancement has been the development of optical coherence tomography (OCT) techniques that use the coherence length of superluminescent diodes to act as a coherence gate to section samples.^1^ This provides a large suppression of multiple-scattering artifacts and allows flows of complex fluids to be spatially resolved from up to 3 mm thicknesses of the specimen. Furthermore, Doppler OCT measurements allow the direct measurement of the velocities of picoliter quantities of complex fluids.^2,3^ To achieve a signal, light must be scattered directly back in the direction from which it originates, i.e., the fluid must have some opacity. The axial resolution of OCT velocimetry is determined by the coherence length and is of the order 9 μm, comparing favorably with similar techniques such as ultrasound, NMR, and photon correlation spectroscopy velocimetry.^4,5^ It also has the advantage over particle imaging velocimetry in that it can be used with opaque fluids without the need for seeding the fluid with particles.

In previous publications, we demonstrated optical coherence tomography (OCT) velocimetry of complex fluids sheared inside a fluid rheometer using infrared light at 1310 nm. Velocity profiles could be measured under shear (e.g., to observe plug flow, wall-slip, and shear banding^4,6−11^), and the intermittency of the velocities could be characterized (e.g., to explore the phenomenon of elastic turbulence^9^). Our bespoke OCT velocimetry apparatus functioned well with a wide variety of opaque complex fluids including DNA solutions,^9^ model hard-sphere colloidal suspensions,^6,8^ tomato ketchup,^12^ shampoo,^12^ margarine,^6^ chocolate,^12^ starch,^4^ and lamellar gel networks^10^ (LGNs, found in hair conditioners and pharmaceutical creams). In the current paper, we have optimized our Doppler OCT measurement apparatus for use on a test rig in chemical engineering at the University of Manchester. This has allowed us to characterize the flow behavior of LGNs and milk powder solutions during in-line processing, thus demonstrating the viability of OCT for monitoring the flow of opaque complex fluids.

The characterization of our fluids was achieved through the measurement of both time-averaged velocity profiles and transient velocity fluctuations. In horizontal and cylindrical pipes, laminar flow is defined by the radial velocity profile. The exact form of this profile depends on the physical properties of the fluid, allowing such a measurement to be used to infer those properties. For a Newtonian fluid in a full pipe, the velocity profile has a parabolic form and is given by the well-known Hagen–Poiseuille (HP) equation,^13^
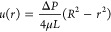
1where *u* is the flow-wise velocity, *R* is the pipe radius, *r* is the radial position in the pipe, μ is the fluid’s dynamic viscosity, and Δ*P* is the change in pressure over the length, *L*, of the horizontal pipe. Non-Newtonian fluids often have a velocity profile with a flattened center when compared with the parabola of HP flow, due to the shear gradient inherent in the pipe geometry. Examples of this include thixotropic fluids,^14^ Bingham fluids,^13^ and shear-thinning power law fluids like LGNs.^10,15,16^ The constitutive equation for such fluids has the form,
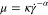
2where γ̇ is the shear rate, α > 0 is the scaling exponent, and κ is a constant of proportionality. The resulting velocity profile is given by,^13^
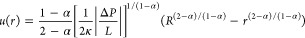
3

In an industrial pipeline, there are many factors that could affect the dynamics of flow including pipe geometry, pump effects, and flow stability. Turbulence is of particular interest as it can be either desirable, e.g., to aid mixing, or disadvantageous due to the larger forces required to drive flow. The ability to detect turbulence in real-time is then potentially very useful. The onset of turbulence is usually characterized using the Reynolds number, *Re* = *LU*ρ/μ, where *L* is the characteristic length scale of the flow geometry, *U* is the bulk flow velocity, and ρ is the fluid density. Laminar HP flow is theoretically stable to infinitesimal perturbations up to at least *Re* = 10^7^.^17,18^ However, finite-amplitude perturbations are hard to suppress, so the transition to turbulence occurs at much lower *Re* due to imperfections in the pipe surface or entrance effects.^19^ The transition is typified by the intermittent appearance of localized turbulence with a finite lifetime, known as *puffs*, at values of *Re* as low as 1500.^17^ Recent experiments have indicated that the critical *Re* for puffs to become self-sustaining occurs at *Re*_*c*_ ≈ 2040.^18,20^

Industrial pipelines are far from the idealized systems of theory or fundamental studies on turbulence. There are a multitude of factors that can have conflicting effects on the flow. For example, extensible polymers and suspended particles are common components in a variety of industrial fluids, and both sensitively affect the transition to turbulence by providing a competing pathway to instability and suppressing intermittency.^21−23^ Pulsatile flow, such as that generated by positive displacement pumps used on many industrial rigs, has also been found to decrease,^24,25^ increase,^26^ and have no effect^27^ on the value of *Re*_*c*_, depending on the exact realization of the flow.

Though the exact value of the velocity at a given point in a turbulent flow is impossible to predict, there are some statistical properties that are universal for fully developed, homogeneous turbulence, such as the histograms of the velocities or velocity fluctuations.^28^ The kinetic energy spectrum for velocity at points separated by a distance *l* is also known to show power law scaling with an exponent of −5/3.^28^ Using Taylor’s frozen hypothesis,^29^ this can be extended to the time fluctuations in velocity at a given point in space, so long as the amplitude of the turbulence is small. OCT allows direct measurement of these transient velocity fluctuations. Applied to an industrial pipeline, this could provide key insights into flow dynamics when the behavior is hard to predict.

The work presented here constitutes the first application of OCT velocimetry to the flow of complex fluids in a cylindrical pipe for in-line processing. The laminar flow of LGNs based on cetyltrimethylammonium chloride (CTAC) and ceto-stearyl alcohol with varying NaCl concentrations and a Newtonian solution of milk powder in water will first be studied using time-averaged velocity profiles. Measurements of the transient velocity fluctuations in both fluids are then used to show stable flow behavior, the effects of dynamic changes to formulation in the LGNs, and flow instability in the milk. We demonstrate that the noninvasive nature, cheap cost compared to other velocimetry techniques, and competitive time and space resolution mean that OCT velocimetry has great potential for in-line monitoring of the flow of opaque fluids in industrial pipelines.

## Materials and Methods

### Pipeline

**Figure 1:**
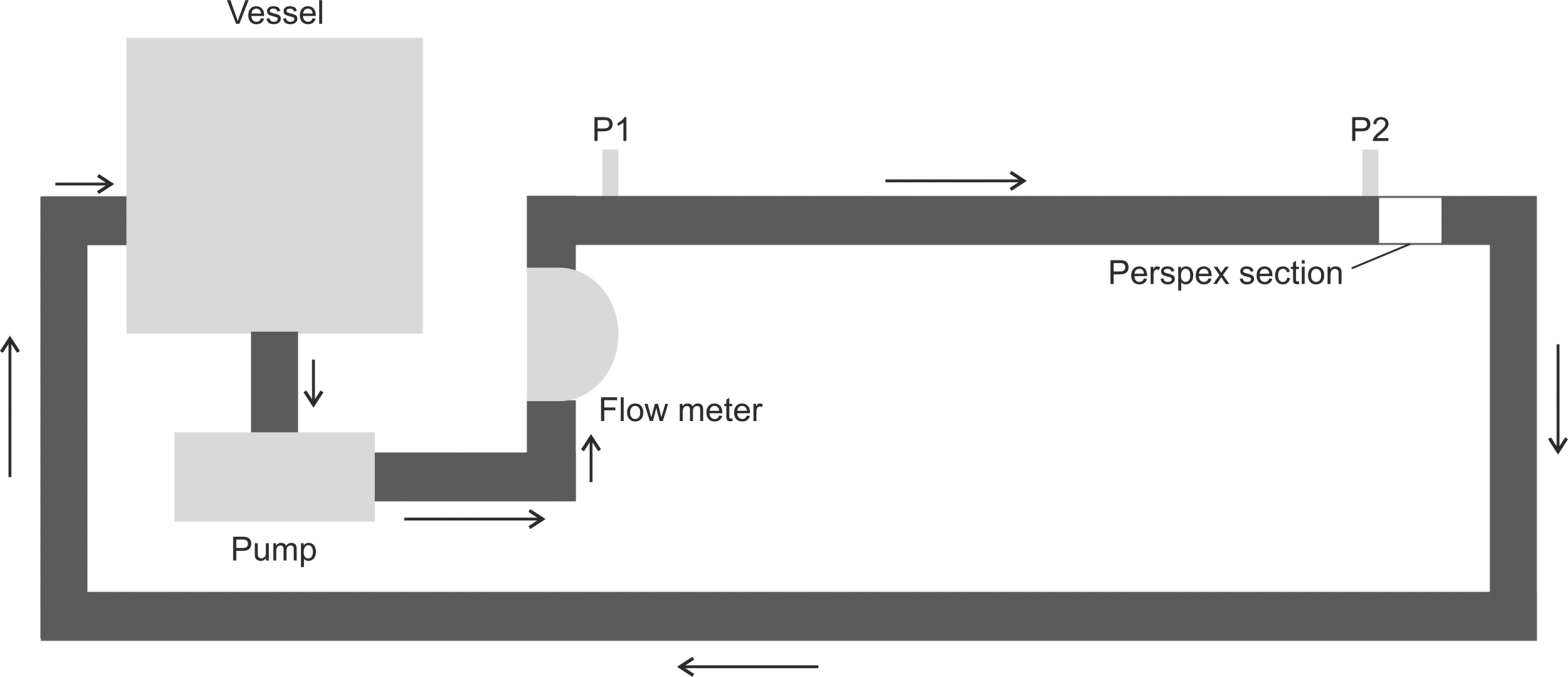
Schematic diagram of the pipe system, with arrows indicating the direction of flow. Fluid is pumped from the bottom of the vessel, travels vertically though the Coriolis flow meter, then comes to the 3 m horizontal pipe. P1 and P2 are the positions at which pressure measurements are taken, while the optical coherence tomography (OCT) velocimetry measurements are performed in the perspex section. The fluid then travels down before returning to the vessel from ports in the side. A more detailed view of the OCT probe and perspex section can be seen in Figure 2.

All experiments in this study were conducted on a skid pipeline in chemical engineering at the University of Manchester. This consists of a 65 L mixing vessel connected to a horizontal pipe with a diameter of 32 mm and a length of ∼3 m. A schematic diagram is shown in Figure 1. In-line measurements of mass flow rate and density are made by a Micro Motion R100S Coriolis flow meter, and the differential pressure is measured at positions 2.77 m apart on the horizontal pipe. The fluid is pumped by a Fristam FKL25 positive displacement pump. This has two rotors with two lobes each, meaning each rotation brings four spikes in pressure, and the flow produced is pulsatile.

### OCT Velocimetry

The short coherence length, *l*_*c*_, of the superluminescent diode (SLD) light source used in OCT means that the condition for obtaining a signal is δ*l* < *l*_*c*_, where δ*l* is the difference in optical path length traveled by the light in each arm of the interferometer.^30^ Any scattered light outside this range does not contribute to the interference pattern that makes up the signal, reducing the impact of multiple scattering and allowing opaque samples to be used. The sample must be sufficiently opaque in the wavelength range of the light source to produce laser speckle for a signal to be achieved. Changing the path length of the reference arm of the interferometer, e.g., via a scanning mirror, allows ∼3.4 pl volumes of sample to be selected for measurement.^8^ The longitudinal resolution is defined by *l*_*c*_, while the transverse resolution depends on the waist of the focused beam.^30^ The maximum depth within the sample is limited by multiple scattering, determined by sample turbidity and also by the depth of focus of the lens system used. This is commonly in the range of 2–3 mm. The time-domain interference pattern caused by the Doppler shift due to movement in the sample can be converted to the frequency domain via a power spectral density (PSD) transformation. The Doppler frequency can then be measured via a Gaussian fit to the data, and the velocity, *v*, can be calculated via the equation,^6^
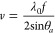
4where λ_0_ is the central wavelength of the light source, *f* is the Doppler frequency (as calculated from the center of the Gaussian), and θ_*a*_ is the angle of the lens relative to the normal of the sample surface. θ_*a*_ is required to avoid backscattering from the surface, obscuring the signal.

**Figure 2:**
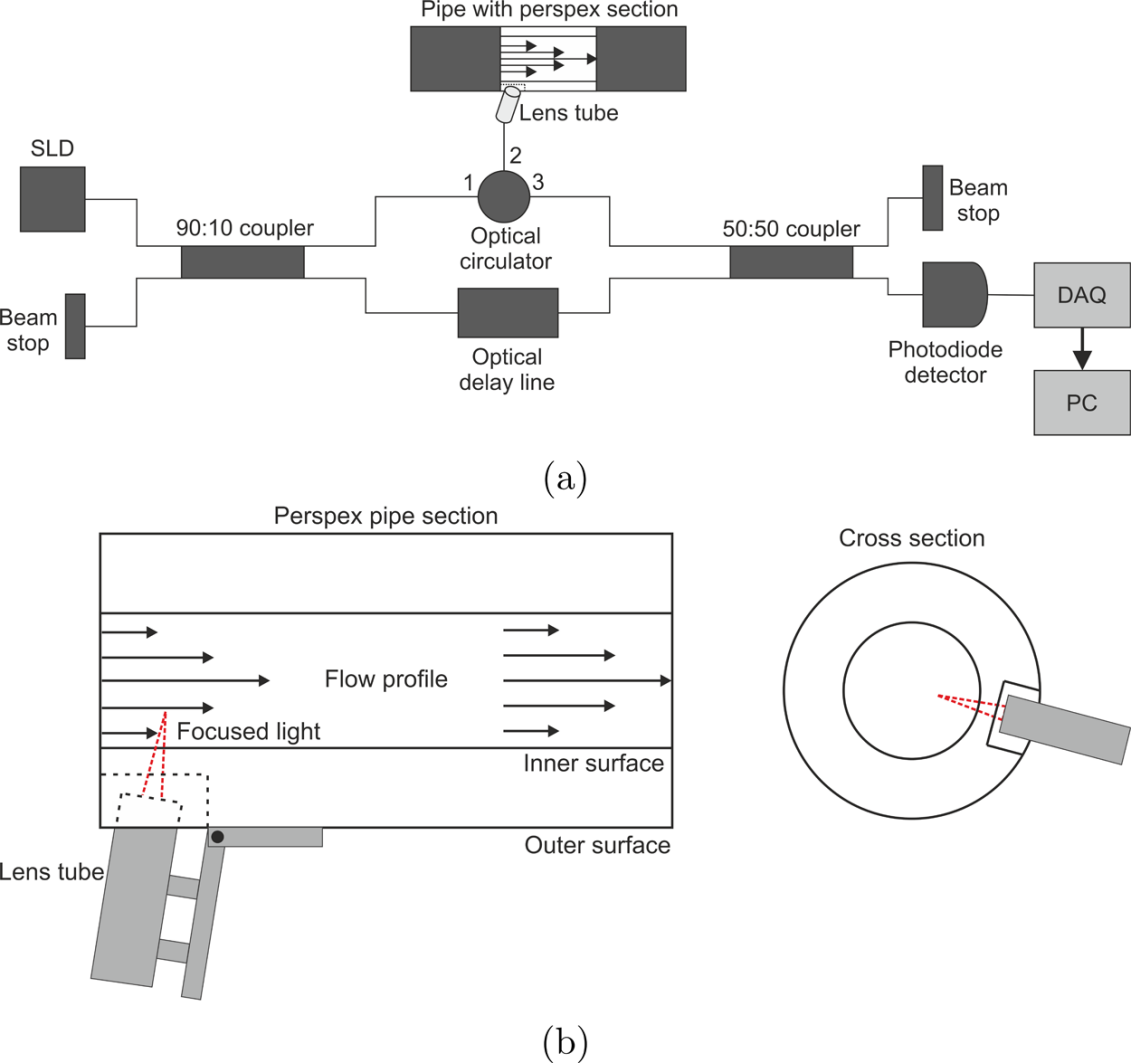
Schematic diagram of the design of the OCT device (a). It is based on a Mach–Zehnder interferometer in which the light in the sample and reference arms are directed down separate paths. The light source is a superluminescent diode (SLD), and each optical component is connected by single-mode optical fibers. The light is directed from the lens tube into the perspex pipe section, located in the pipe system, as shown in Figure 1, to make measurements of the fluid velocity. A more detailed diagram of the perspex section and lens tube from both side on and as a cross-section can be seen in (b). The perspex is machined down to a thickness of 5 mm to create a window into which the lens tube can be inserted to make measurements.

The apparatus used in this study was custom-built based on the time-domain design used by Malm et al.^8^ A schematic diagram of the apparatus is shown in Figure 2a. We wanted to test the feasibility of OCT as a technique for in-line monitoring of industrial processes. Thus, as a first iteration, emphasis was placed on simplicity and robustness. The electro-optic modulator method for reducing 1/*f* used by Malm et al. was removed, and the scanning mirror was replaced by a delay line so that no mirror alignment is necessary, increasing the robustness of the apparatus. The use of a Mach–Zehnder design, where the light in each arm of the interferometer travels down separate paths, was retained to incorporate the boxed optical delay line. The light source used is a Covega 1021 superluminescent diode (SLD) with a 65 nm bandwidth, centered at 1310 nm, and *l*_*c*_ = 9 μm. This is connected to a fiber-based 90:10 coupler that ensures 90% of the light is directed to the sample. This is important as the power returning from each arm must be balanced to produce a signal with optimal fringe contrast, and much of the incident intensity is lost to scattering and absorption within the sample. An additional 15 dB (∼3% transmission) fiber attenuator had to be added to the reference arm to achieve this. In the sample arm, the light is then directed to a three-port optical circulator, which first directs the light to the lens system, into the sample, and then to the final 50:50 coupler. The lens system is made up of a Thorlabs F240APC-C collimating lens and a C280TMD-C focusing lens connected by a lens tube. The reference arm consists of an Oz-optics ODL-650-MC optical delay line, consisting of a mirror mounted on a translation stage operated via a servo motor. A single step of translation corresponds to changing the position of observation within the sample by ∼0.15502 μm if the sample has a refractive index of 1. Once the light is recombined, the signal is measured by a photodiode detector and digitized by a National Instruments USB-5133 device capable of sampling at rates >250 kHz. The LabVIEW program is then used for data acquisition and device control. All components are connected by angle-cut single-mode optical fibers to reduce losses due to backscatter. Unless otherwise stated, each component was purchased from Thorlabs.

The probe used consists of a short length of polished perspex pipe that can be screwed into the skid pipeline. It was machined down to a width of 5 mm in one position, forming a window. An aluminum tube, into which the lens system can be inserted and fixed, has been attached to the outside. The longitudinal angle of the lens system can be altered to reduce the impact of backscatter from the pipe surface, while still ensuring that the beam passes through the pipe center. A diagram can be seen in Figure 2b.

### Working Fluids

There are two working fluids used in this paper. The first is a lamellar gel network (LGN) based on a 3:1 ratio of cetyltrimethylammonium chloride (CTAC) to ceto-stearyl alcohol. This was provided by Unilever and made according to the European patent by Casugbo et al. (2014)^31^ and detailed by Cunningham et al. (2021),^32^ and used previously in work by Watts Moore et al. (2023).^10^ The textural properties of LGNs mean they are commonly found in cosmetics such as creams and hair conditioners.^33^ They are a multiphase structure consisting of a lamellar bilayer gel phase, bulk water, crystals of hydrated fatty alcohol, and potentially oil stabilized by a surfactant monolayer.^33−35^ Despite their ubiquity, the complex nature of the microstructure mean that LGNs display a range of nonlinear rheological behavior that is poorly understood.^10,36−39^ This includes a yield stress,^33,36,40^ wall slip,^10,15,41^ shear banding,^10,15,37,41−43^ thixotropy, and power law rheology.^10,15,16^ To mimic the way in which dynamic formulation changes are made in an industrial setting and study the changes to the flow, NaCl in aqueous solution was added directly to the vessel to increase the salt concentration. Starting with ∼40 kg of LGN, 203.96 g of NaCl in 577.73 g of water, 320.17 g of NaCl in 904.69 g of water, and 341.73 g of NaCl in 943.58 g water were added to the mixing vessel to increase the NaCl concentration to 0.5, 1.25, and 2% w/w in sequence. In each case, the NaCl solution was poured into the vessel of the course of <10 s, with the agitator operating at 50 rpm to aid mixing.

The second fluid was a solution of ∼2.6% w/w skimmed milk powder in water. This was chosen to provide the optical turbidity required for an OCT signal in a Newtonian fluid. The milk powder’s composition was: max 1.25% fat, 31–38% protein, and 48–56% lactose.

## Results and Discussion

### Velocity Profiles

**Figure 3:**
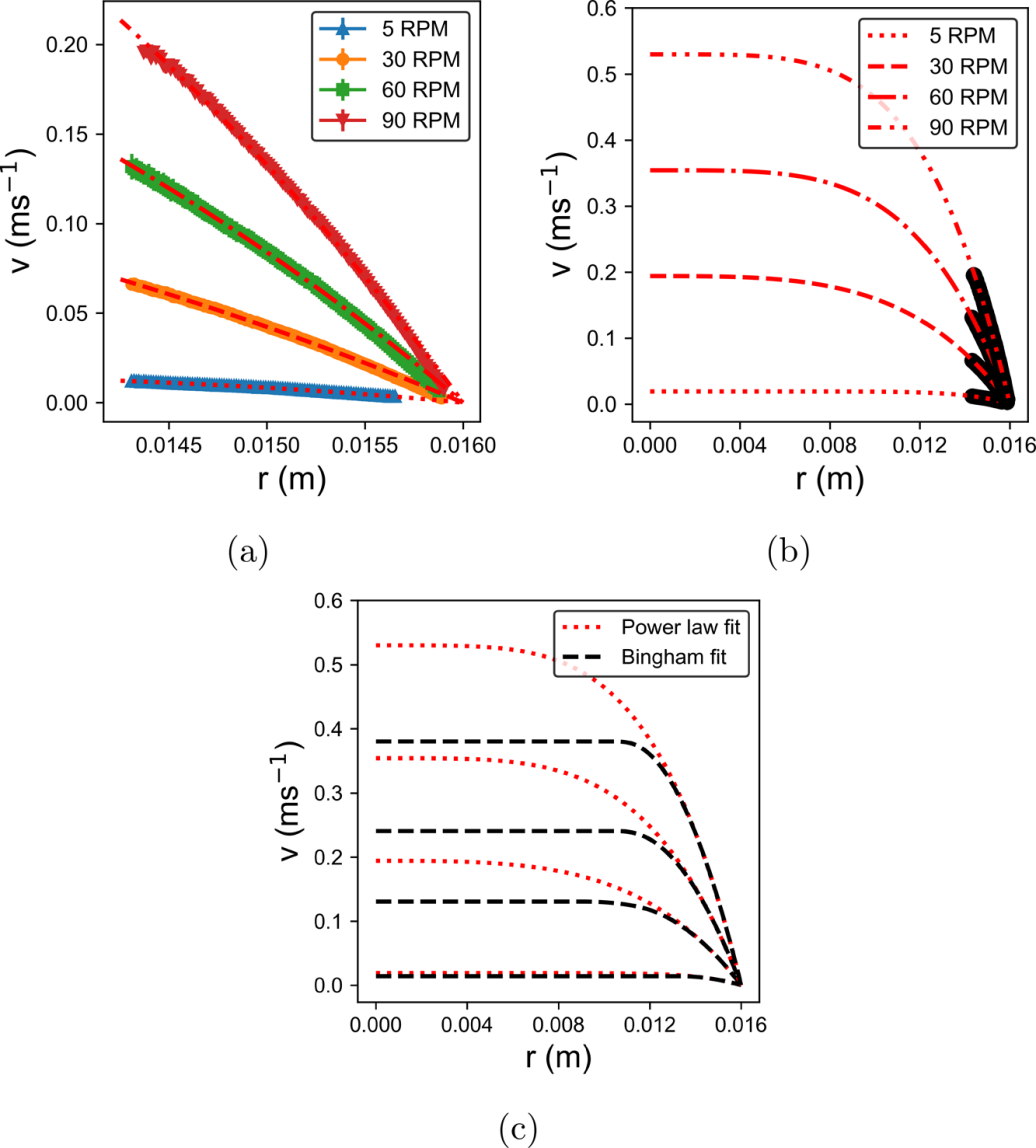
(a) Mean radial velocity profile *v*(*r*) is plotted as a function of radius in the pipe, *r* for pump speeds in the range 5–90 rpm for the LGN sample with no salt with the mean power law fit based on eq 3 overlaid as a dashed line. The fits are extrapolated to the pipe center in (b). A comparison of power law and Bingham fluid fits can be seen in (c), showing convergence close to the wall and divergence of the velocity plateau.

Lamellar gel networks, such as the one studied in this paper, have been shown to exhibit behavior associated with soft glassy rheology.^10,15^ One feature of soft glassy rheology is power law scaling of viscosity with shear rate.^44^ In the simplest case, where there is no yield stress, this is of the form shown in eq 2. The resulting velocity profile in pipe flow is given by eq 3.^13^ Though our lamellar gel network is strongly thixotropic with a long relaxation time and has a yield stress associated with this,^10^ after sufficiently long times and at high rates, it is well-approximated by eq 2. As such, we have chosen to fit curves of the form of eq 3 to our profiles in order to determine the character of the steady-state flow. The mean velocity profiles of the LGN with 0% NaCl can be seen in Figure 3a, with the mean plug flow fits superimposed as dashed lines. The final fits, extended to the pipe center, can be seen in Figure 3b, revealing the characteristic velocity plateaus.

**Figure 4:**
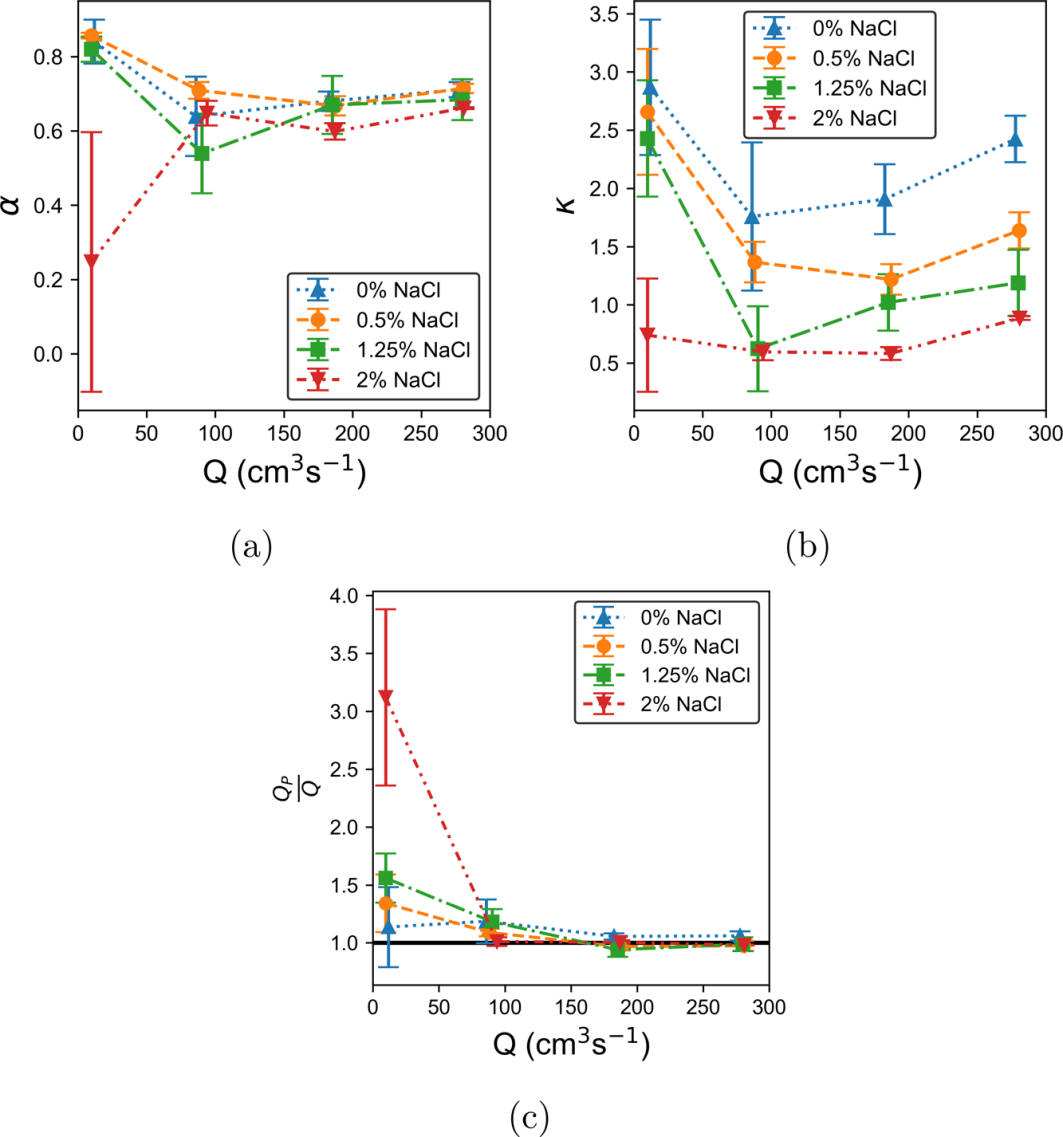
Mean power law exponent, α, constant of proportionality, κ, and ratio of fit flow rate to measured flow rate *Q*_*P*_/*Q* are plotted against measured volume flow rate, *Q*, for all salt concentrations with LGNs in (a–c), respectively. The solid line at *Q*_*P*_/*Q* = 1 in (c) is included to draw attention to the point at which the extrapolated flow rate following eq 5 is equal to the measured quantity.

OCT can only probe depths of ∼2 mm into the pipe, which does not extend into the low-shear plateau. The extrapolated fit thus does not prove the existence of the plateau. Reasonable fits to the data can also be made using the velocity profile for a Bingham fluid that is Newtonian above a yield stress, as can be seen in Figure 3c. It is therefore important to compare the fit parameters to independently measured properties for verification. The fit parameters α and κ for each NaCl concentration can be seen plotted as a function of pump rate in Figure 4a,b. The volume flow rate for the plug flow fit, *Q*_*P*_, can be calculated from these parameters via the flux integral of eq 3. This is given by^13^
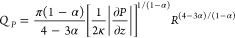
5For a given power law fluid, it is expected that α and κ should remain constant with the pump rate. In the range of 30–90 rpm, this is approximately true for α, while κ seems to show slight rate dependence. In both cases, the values at 5 rpm are significantly different. This is likely to be a result of the fluid's thixotropy. At low rates, it takes longer for the fluid to reach steady state. The internal stresses will also be closer to the yield stress than at higher rates, meaning the fluid may be acting like a Herschel-Bulkley fluid rather than a pure power law fluid. The 5 rpm parameters for the 2% NaCl solution show significant differences again from their lower concentration counterparts. This is due to the pipe becoming partially full. The partial filling of the pipe was an unexpected limitation of our experimental setup and appears to be related to the pump speed and fluid viscosity. High-viscosity fluids, such as the LGN, can fill the pipe at low pump speeds, while the milk solution required a pump speed of 70 rpm to achieve a full pipe. Figure 4b shows that for a given shear rate, the viscosity of the LGN (via κ, the prefactor) is negatively correlated with NaCl concentration. At 2%, this has become significant enough for a small air gap to appear at the top of the pipe. This means that the velocity profile and flow rate in eqs 3 and 5 no longer apply in their given forms,^45,46^ and the measurement of the differential pressure used in the calculation of α and κ may have become inaccurate.

Figure 4c shows how the ratio of *Q*_*P*_/*Q* varies with pump rate for each salt concentration, where *Q* is the mean flow rate measured directly by the in-line Coriolis flow meter. From 30 rpm, this starts to collapse to unity, indicating that a power law velocity profile is an appropriate model when the pump rate is not at low pump rates. In contrast, using a Bingham model produces an underestimation of the flow rate. The discrepancy between the data and the fit close to the wall is negligible in this calculation, amounting to a reduction of ∼0.25%, well within the error bars of *Q*_*P*_/*Q*.

**Figure 5:**
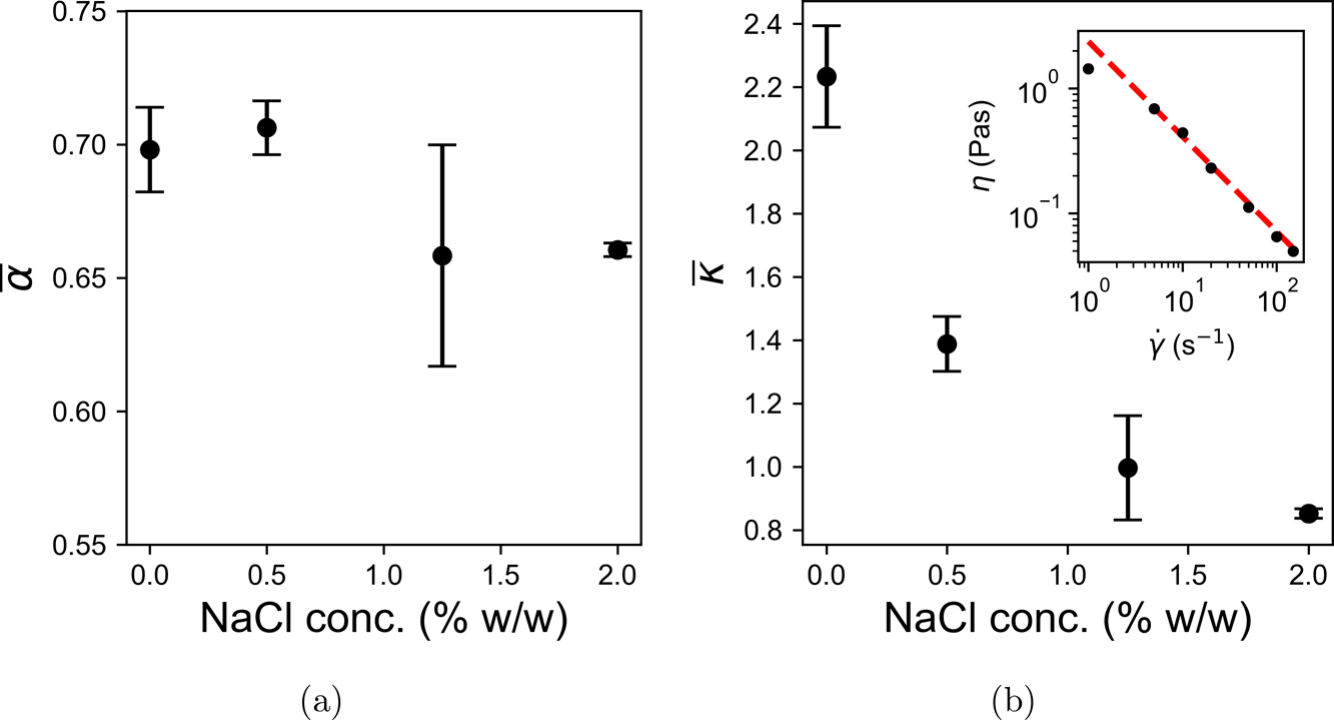
Figures (a) and (b) show the weighted mean of the power law exponent, α̅, and constant of proportionality, κ̅, from eq 2 against salt concentration for LGNs. Only fit parameters from fully developed flow, i.e., rejecting those at 5 rpm, have been included in this calculation. The inset to (b) shows the asymptotic instantaneous viscosity, η, as a function of shear rate γ̇ for a conditioner formulation with the same surfactant ratio. The original data can be found in Figure 2b of Watts Moore et al. (2023).^10^ A power law fit with the parameters α_*rh*_ = 0.76 ± 0.03 and κ_*rh*_ = 2.4 ± 0.2 is overlaid as a dashed line.

The mean values of α and κ (α̅ and κ̅) for pump rates in the range of 30–90 rpm are plotted against the NaCl concentration in Figure 5a,b. The calculation of each mean was weighted by the uncertainty on each value to give less prominence to those results with large variance. The dependence of α on concentration appears to be weak, with a modest decrease occurring with increased concentration. In contrast, κ shows a robust inverse relationship with the pump rate, meaning the viscosity at a given value of γ̇ decreases as salt concentration increases.

The inset of Figure 5b shows the relationship between the instantaneous viscosity and shear rate for an LGN with an identical formulation to the one used in this paper. This was collected by shearing a sample in a rotational shear rheometer with a 40 mm diameter parallel plate geometry and a gap size of 500 μm at a constant shear rate for 1000 s. The mean viscosity in the final 10 s was then calculated and plotted against γ̇. The full raw data can be found in Figure 2b of Watts Moore et al. (2023).^10^ The fluid viscosity does not reach a plateau within the 1000 s, but by the end, the absolute viscosity reduction has slowed enough that the percentage uncertainty on each mean value is ≲0.5%. A power law fit to these data produces the parameters κ_*rh*_ = 2.4 ± 0.2 and α_*rh*_ = 0.76 ± 0.03. The mean results from the velocity profile fit parameters for 0% salt are κ̅ = 2.2 ± 0.2 and α̅ = 0.70 ± 0.02. These results are compatible within the 95% confidence interval, despite the fact that ∼0.6 mL of fluid was used in the rheometer experiments compared with ∼40 L in the pipeline, with completely different flow geometries.

**Figure 6:**
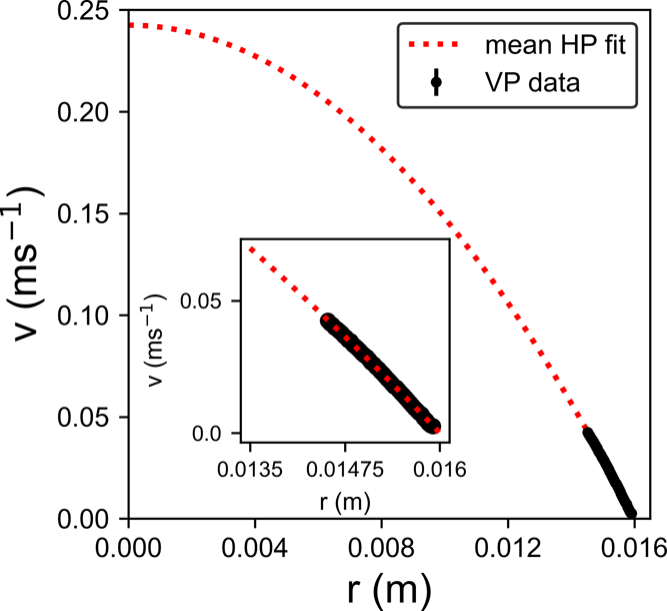
Mean radial velocity profile for milk pumped at 30 rpm with a parabolic Hagen–Poiseuille profile fit using eq 1 for a Newtonian fluid. Close up of the fit to data shown in the inset.

Figure 6 shows the velocity profile close to the wall for the solution of milk powder in water. At this shear rate, the pipe was half-full, meaning the expected profile for a Newtonian fluid is given by eq 1.^45,46^ The mean fit to three data sets is shown as a red dotted line in Figure 6 and, assuming a half-filled pipe, gives a flow rate ratio of *Q*_*HP*_/*Q* = 1.337 ± 0.006, where *Q*_*HP*_ is the mean volume flow rate obtained from the fit to data. There were several challenges involved with getting an accurate measurement here, which may have resulted in the deviation of *Q*_*HP*_ from *Q*. The lower viscosity of the milk compared with the conditioner caused the pulsatile nature of the flow to be more apparent and the flow less steady. For pulsatile flow in rigid pipes where there is no reversal of mean flow direction, the velocity profile of a Newtonian fluid is the superposition of HP flow and a time-dependent component dependent on the pressure gradient.^47,48^ The instantaneous velocity profile will be changed from its classic parabolic shape, but any time-averaged quantities over several cycles will be identical to those obtained from HP flow.^48−50^ The data for each point of the velocity profile were taken over 6.5 s. For a pump speed of 30 rpm, this means that ∼13 cycles of the pulsating pressure gradient are included in the average and should not contribute to the shape of the velocity profile. The fill level of the pipe is also not known accurately, possibly introducing a systematic error into the calculation of *Q*_*HP*_. The partial filling means that the pressure drop measurements taken here are inaccurate, meaning that a value for the viscosity cannot be estimated. However, the local velocity measurements made using OCT are unaffected. As long as the fill level is acknowledged, the velocity profile of a laminar fluid measured using OCT velocimetry can still be used for characterization using an adjusted equation^45^ in the plausible real-world scenario of a partially full pipe.

The choice of milk as a Newtonian counterpart to the conditioner in this study was also hampered by aging produced by biofouling. This is the combination of several processes, both chemical and biological in origin, that can cause complicated changes to the flow properties of the milk.^51^ Ting et al. (2016)^51^ found that the same sample of milk can undergo both decreases and increases in viscosity at different times in the aging process. Aging was evident in our experiments, with the milk visually becoming less optically turbid and eventually solids being produced. This had an effect on experiments, as the flow rate response to a given pump speed was not consistent. This took the form of long-time oscillations with a frequency that is a decreasing function of pump speed in single experiments and a tendency for the average flow rate at a given pump speed to increase as time went on.

### Transient Velocity

**Figure 7:**
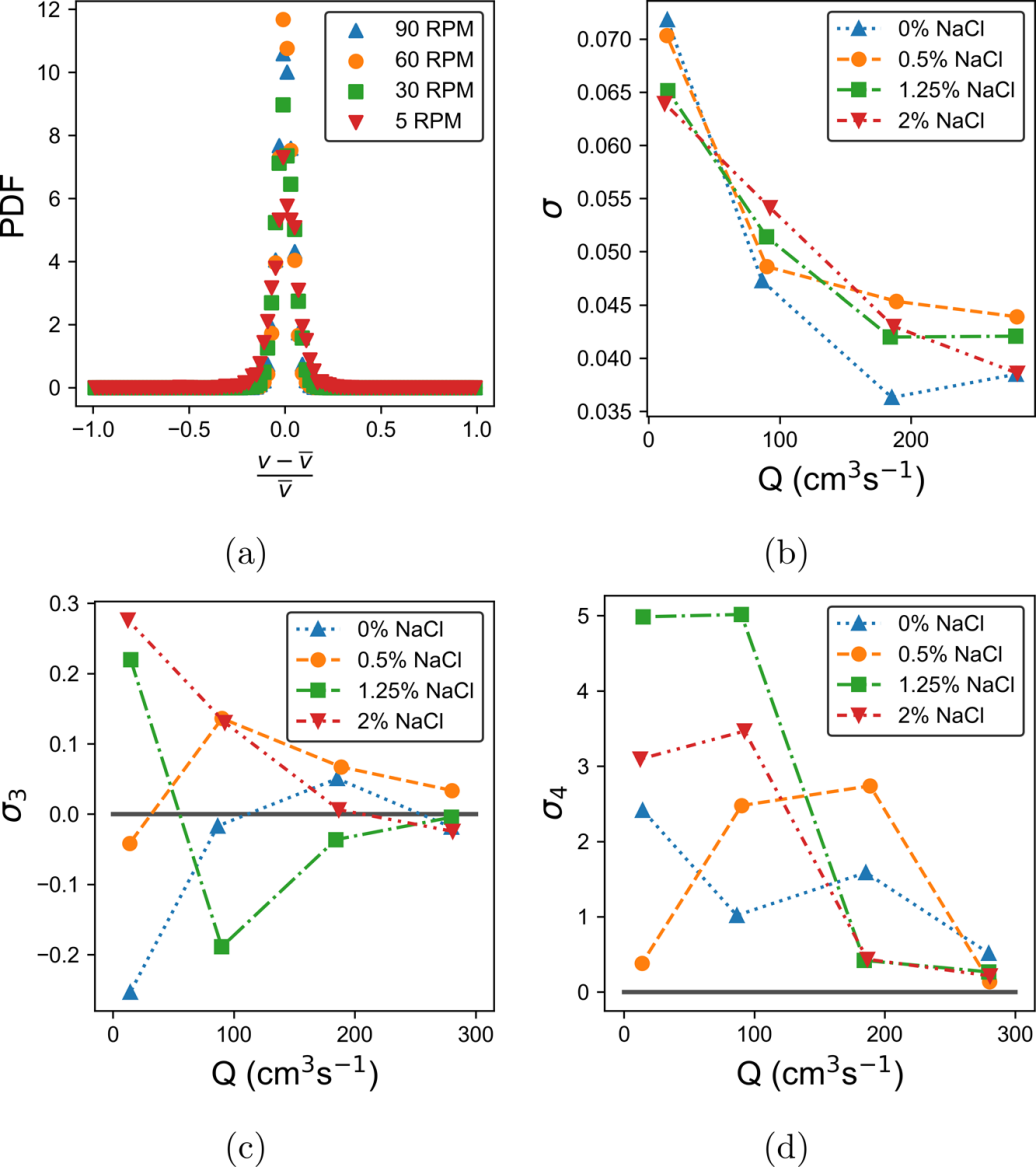
Plot of the probability density functions (PDFs) of the velocity fluctuations, (*v*–*v̅*)/ *v̅* for transient velocity measurements of the LGN with 0% salt at pump rates from 5 to 90 rpm (a). The standard deviation, skew, and kurtosis of the PDFs of (*v*–*v̅*)/ *v̅* for each salt concentration can be seen in (b), (c), and (d) respectively. A line has been drawn at zero in figures (c) and (d) to aid the eye.

**Figure 8:**
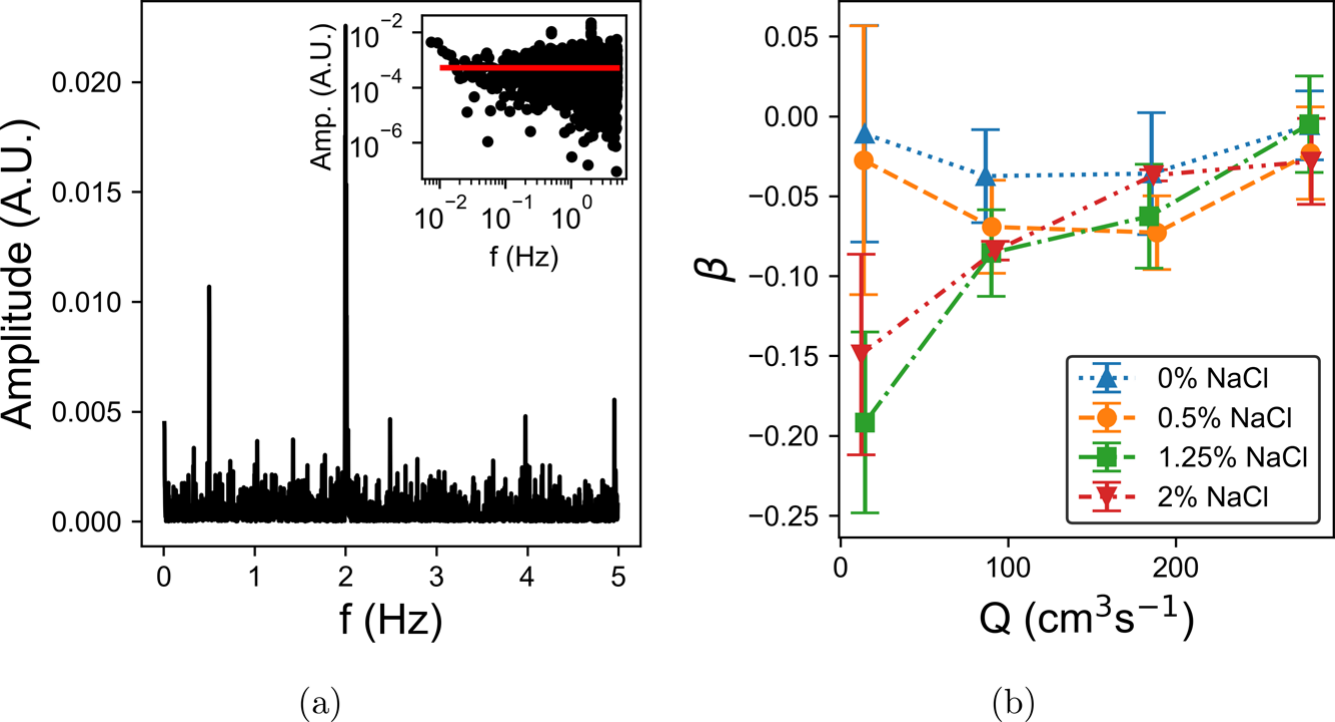
(a) Power spectral density (PSD) of transient velocity fluctuations in the LGN with 0% salt, pumped at 30 rpm. The large peaks at 0.5 and 2 Hz correspond to the frequency of the pump. The inset shows the same data on log scales with a power law fit. (b) Mean power law exponent, β, is plotted against the pump rate for each concentration. The exponent is very low in all cases, indicating stable flow.

The stability of flow can be investigated by studying the statistics of velocity fluctuations, (*v*–*v̅*)/*v̅*. Here, *v* = *v*(*t*) is the velocity at a given time for a fixed position, and *v̅* is the mean velocity measurement at that position. Laminar flow of a Newtonian fluid is expected to produce Gaussian statistics, but flow instabilities can produce deviations from this. Figure 7a shows the probability density functions (PDFs) for the LGN with 0% salt, pumped at rates from 5 to 90 rpm. Figure 7b–d shows how the standard deviation, σ, skew, σ_3_, and kurtosis, σ_4_, of the PDFs vary for all formulations. All transient velocity data sets for LGNs were taken at a position 740 μm from the pipe wall. Each formulation behaves similarly, with σ reducing to a plateau with pump rate and the distribution becoming closer to Gaussian, i.e., σ_3_ and σ_4_ tend to 0. The deviation from Gaussian statistics seen at low rates here is unlikely to indicate flow instability due to the viscosity of the fluid. Instead it is likely to stem from the flow evolving due to thixotropy. At higher rates, the fluid reaches steady state more quickly. Figure 8a shows the power spectral density of the velocity fluctuations for the 0% salt LGN pumped at 30 rpm. There are many peaks present in the spectrum, which is quite noisy owing to the pulsatile nature of the pump driving the flow. The largest peak occurs at 2 Hz, corresponding to the pump frequency multiplied by four. This results from the four-lobed geometry of the pump, causing four spikes in pressure per rotation. The scaling of the kinetic energy spectra of fluids can reveal the stability of the flow. If the flow is stable, a flat distribution will be produced. An unstable flow instead gives a decreasing power law. The exponent depends on the character of instability. For example, an elastic instability produces an exponent, β < –3^21,52^ (observed previously for semidilute DNA solutions^9^), while inertial turbulence follows Kolmogorov scaling with β = –5/3.^28^Figure 8b shows how the exponents of spectra of each LGN vary with the rate. In all cases, β remains close to zero, as would be expected given the well-defined velocity profiles and the high viscosity of the fluid in question.

**Figure 9:**
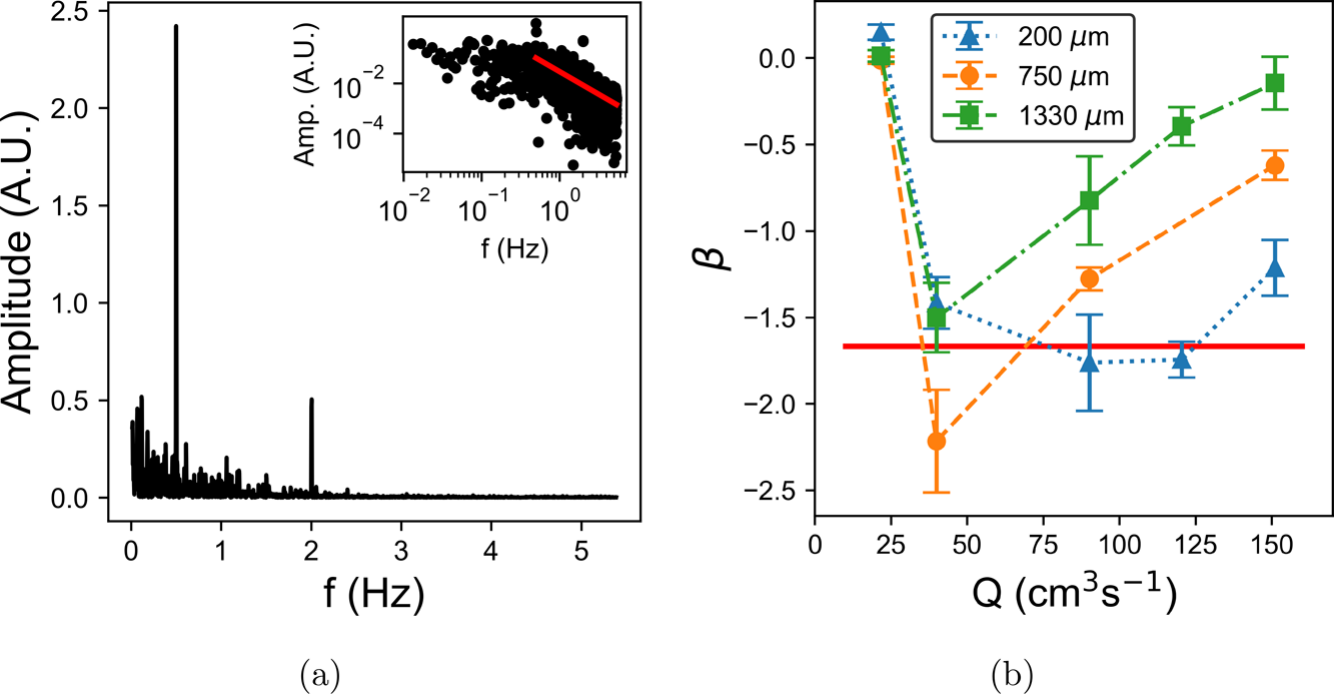
Power spectral density (PSD) of a transient velocity fluctuations at a position of 750 μm in milk pumped at 30 rpm (a). Large peaks at 0.5 and 2 Hz can again be seen corresponding to the pump frequency. The inset shows the same data on log scales with a power law fit to the decreasing section. The mean power law exponent, β, in the decreasing section is plotted against pump rate at three positions in the pipe in (b). A red line at the Kolmogorov scaling exponent of −5/3 for turbulent flow^28^ is included to aid the eye.

Figure 9 shows the power spectral density (PSD) of the velocity and power law exponent (β) for milk, again pumped at 30 rpm. At every pump speed above 20 rpm, the velocity appears to be unstable, and the PSD of the fluctuations includes a region with a power law decrease, e.g., the inset to Figure 9a. A power law was fit to each spectrum, taken at three positions in the pipe. The exponents are shown in Figure 9b. This is in contrast to the laminar behavior shown at 30 rpm in Figure 6, resulting from physical changes to the milk produced by biofouling. The most prominent peaks in Figure 9a occur at 0.5 and 2 Hz, corresponding to the pump frequency and four times this value, respectively. In contrast to Figure 8a, the pump frequency itself is the larger peak. This is because the 4× frequency peak lies in the decreasing power law region of the spectrum and is suppressed by the turbulent dynamics. At 20 rpm, the exponents at each depth are close to zero, indicating that the flow is stable and laminar. At 200 μm from the pipe wall and pump speeds greater than 20 rpm, β becomes close to the –5/3 value expected for inertial Kolmogorov turbulence. This exponent is only expected in situations where the turbulence is both homogeneous and isotropic (i.e., fully developed),^28^ and transitional regimes typified by intermittency are known to cause deviations from it. It then seems strange to see it appear so sharply in response to a relatively small increase in pump speed. When studying turbulence in partially filled pipes or open conduits, it is common to use the hydrodynamic diameter, *D*_*H*_, as the characteristic length scale in the calculation of the Reynolds number. This is defined in terms of the cross-sectional area of the fluid, *A*, and the wetted perimeter of the channel, *P*_*w*_: *D*_*H*_ = 4*A/P*_*w*_. Ng et al. 2018^46^ also suggested using the equivalent diameter of a full circular pipe with the same fluidic example, *D*_*EQ*_ = 2(*A*/π)^1/2^, to facilitate comparison with fully filled pipes. This leads to the definitions of the hydraulic Reynolds number, *Re*_*H*_, and the equivalent Reynolds number, *Re*_*EQ*_:
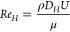
6a
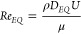
6b

We do not have accurate pressure readings due to the partial fill of the pipe, and the aging had an unknown effect on the viscosity. As such, we do not have an accurate estimate for μ. Taking the viscosity of water, μ_water_ ≈ 0.001 Pa s, as the lower limit of viscosity gives upper limits for the Reynolds numbers at 30 rpm of *Re*_*H*_, *Re*_*EQ*_ = 2673, 1922. This suggests that the flow at 30 rpm should exist in the transitional regime between intermittent puffs and fully developed turbulence. Solid aggregates were seen to form, meaning that the fluid had become a polydisperse suspension. The presence of monodisperse particles in pipe flow can cause a transition to turbulence at Reynolds numbers as low as *Re* = 800, while suppressing the intermittent regime at high enough volume fractions.^23^ The effects of polydisperse particles on turbulence have not yet been studied, and the particle concentration and size distribution remain unknown in our case. Whether the turbulence produced via particle-laden flow should show the same 5/3 scaling in the energy spectrum is another open question. For example, elastic turbulence produces similar suppression of intermittency and changes to *Re*_*c*_ and does not share the same scaling exponent as Newtonian turbulence.^9,21,52^ However, it should be noted that while the friction factor for particle-induced turbulence tends to a similar value to the Newtonian case,^23,53^ elastic turbulence produces completely different results.^22^ This highlights the need for further research on the onset of turbulence in complex fluids.

**Figure 10:**
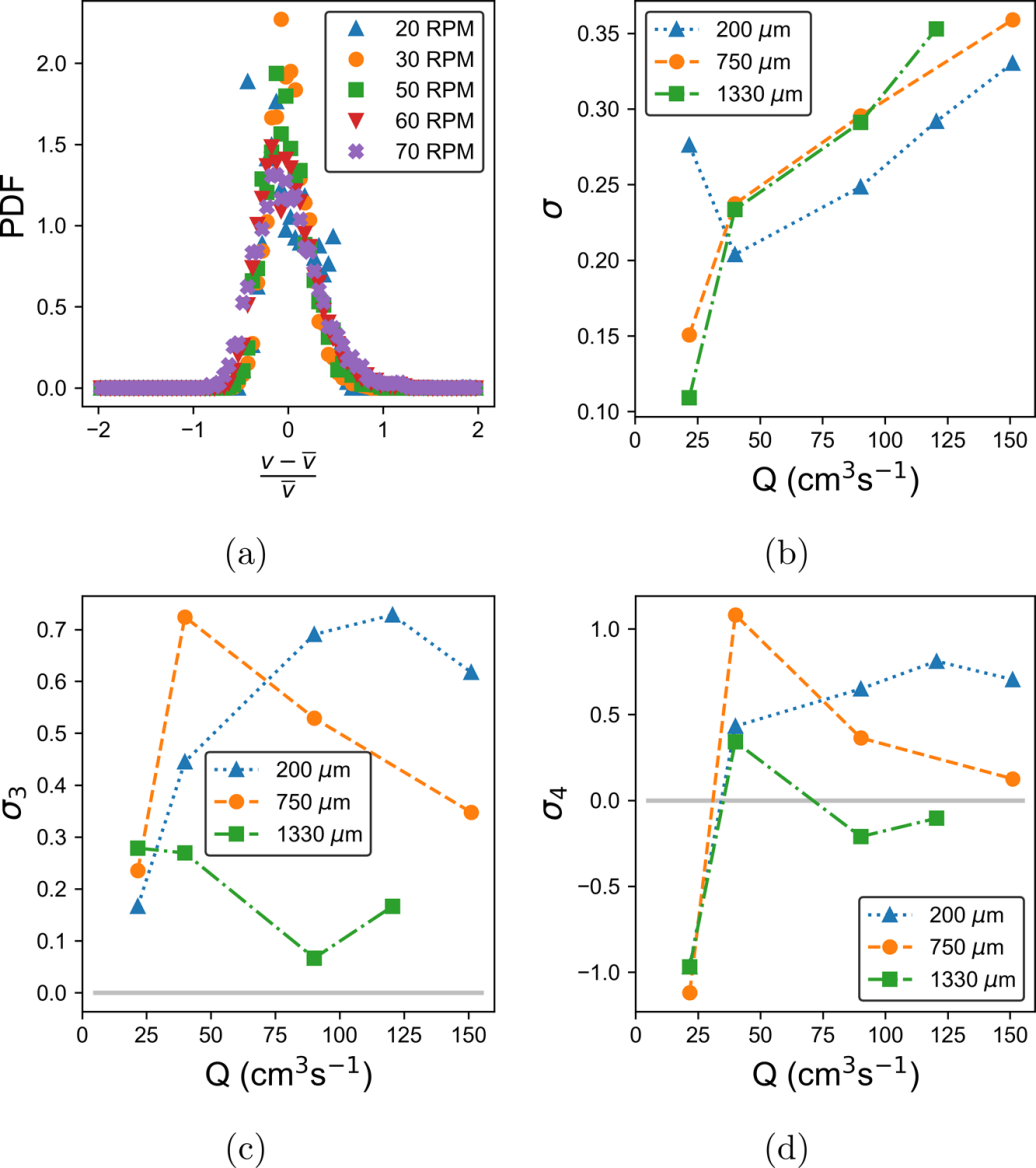
PDFs of the velocity fluctuations of milk at a position of 200 μm from the pipe wall (a). (b) Standard deviation, (c) skew, and (d) kurtosis at depths of 200, 750, and 1330 μm for the velocity fluctuation distributions.

From Figure 9b, there is a clear trend away from β ≈−5/3 at an increased distance from the pipe wall and increased flow rate. There are several possible reasons for this. Figure 10b shows that the magnitude of the turbulent velocity fluctuations increases with both depth and pump speed. This is significant for two reasons. First, it could negate the validity of Taylor’s frozen hypothesis and the equivalence of the energy scaling in time and space. Second, the large velocity fluctuations, combined with the relatively low turbidity of the sample in question, reduced the signal-to-noise ratio. This resulted in gaps in the velocity fluctuation time series, affecting the results of the PSD. Alternatively, the deviations from 5/3 scaling may just stem from intermittency in the turbulence. However, this would not explain why, taking the 1330 μm curve as an example, β is close to −5/3 at 30 rpm, but decreases in magnitude thereafter. Agrawal et al. 2019^23^ found that for particle-laden fluids at intermediate concentrations, as *Re* is increased, the flow would display two transitions to turbulence. The first is a continuous, nonintermittent transition due to the presence of particles, and the second is an intermittent transition typical of Newtonian turbulence. The reductions in the magnitude of β at higher rates could then be an expression of a mixed state such as this. Finally, in many experiments, the adherence to 5/3 scaling is not perfect, and fitting a power law to data is in general sensitive to the choice of fitting region.^28^ These factors make it difficult to draw concrete conclusions about the fundamental nature of the instability on display here. Refinement of the experimental setup to remove unknowns such as the pipe fill level and fluid aging would undoubtedly shed more light on this question, while direct access to the spatial energy spectrum would also remove the reliance on Taylor’s frozen hypothesis. In the case of an OCT apparatus, the latter could be achieved through the use of two fibers with a variable separation capable of simultaneous measurement. However, in an industrial setting, there may be parameters that lead to less-than-ideal conditions, but nonetheless cannot be altered. In such cases, the statistical character and fundamental origin of a flow instability will often be of secondary importance to the physical existence of the instability. Our measurements make it clear that there is a bifurcation in flow behavior between 20 and 30 rpm due to the onset of such an instability, highlighting the potential use of OCT velocimetry for in-line flow monitoring.

Figure 10a shows the PDFs of the velocity fluctuations of milk 200 μm inside the pipe at rates of 20–70 rpm. Compared with the distributions for the LGN in Figure 7a, these PDFs are less symmetric in general. This is related to the instability in the flow from 30 rpm upward and also because the pipe only becomes fully filled at 70 rpm, increasing the possibility of secondary flows.^45,46^ At 20 rpm, when the flow is laminar, the pulsating flow produces a non-Gaussian distribution. There was also a small amount of clipping at 200 μm, as the trough of the velocity oscillations dipped below the noise floor of the measurement. This produced the sharp peak seen on the left extreme of Figure 10a and the increased value of the standard deviation, σ, in Figure 10b. The incorporation of a fiber-based electro-optic modulator into the interferometer design would allow the measurement of smaller velocities, reducing the impact of clipping artifacts and potentially revealing surface dynamics close to the pipe wall. Figure 10b shows that the standard deviation of the velocity fluctuation distributions, in general, increases with the rate. This is in contrast to the behavior in Figure 7b and is indicative of instability in the flow. Figure 10c,d again shows contrasting behavior to the equivalent plots for the LGN owing to the departure from Gaussian statistics expected from turbulent flow^28^ and reiterating the bifurcation in behavior that occurs between 20 and 30 rpm, as shown in Figure 9b.

### Dynamic Formulation

**Figure 11:**
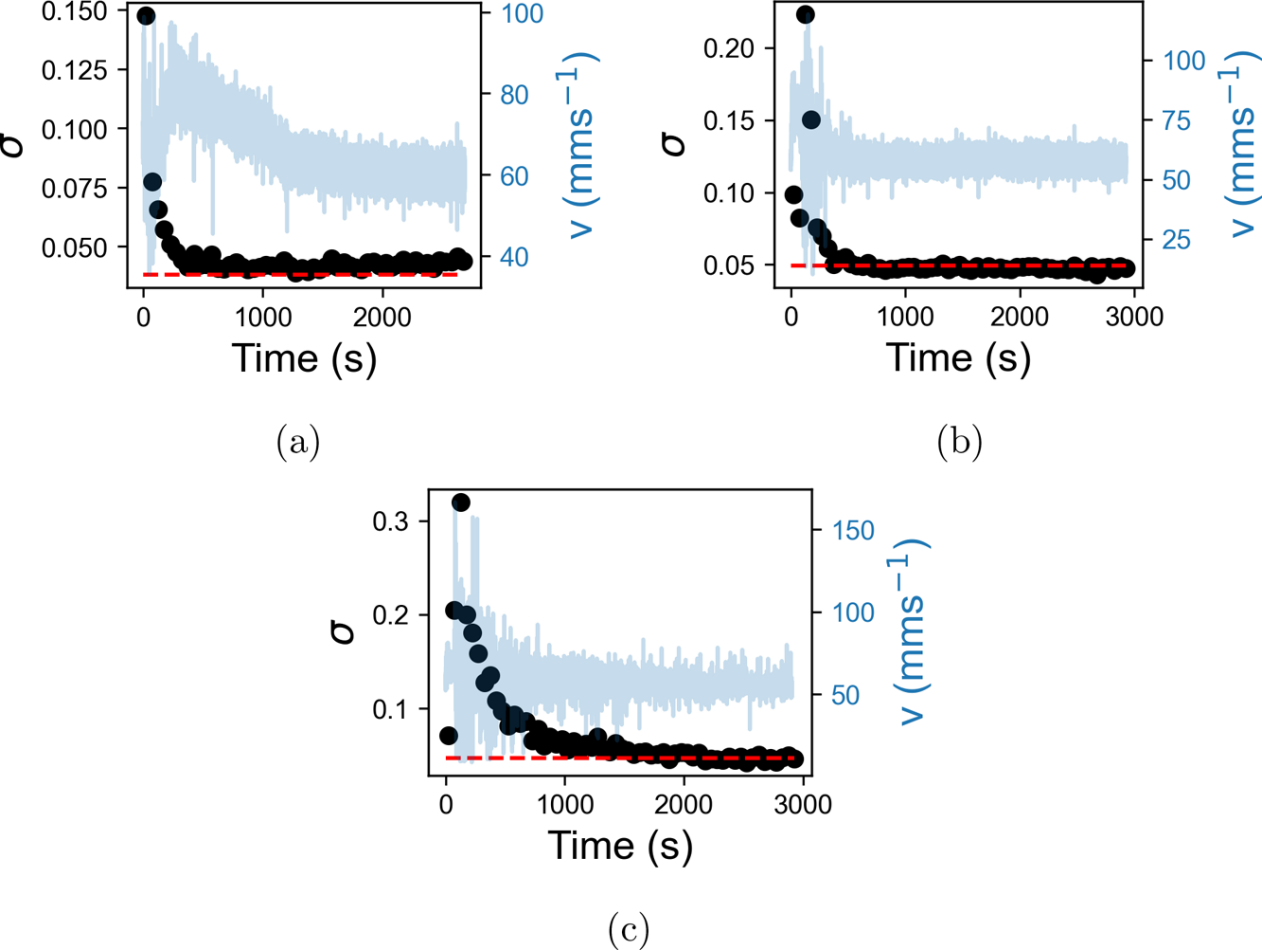
Standard deviation, σ, of the velocity fluctuation distributions of LGNs in 50 s intervals after the addition of salt solution (NaCl). Increases of NaCl to 0.5, 1.25, and 2% are shown in (a), (b), and (c), respectively. The preaddition value of σ is shown on each plot as a red dotted line, and the velocity is plotted in the background.

**Figure 12:**
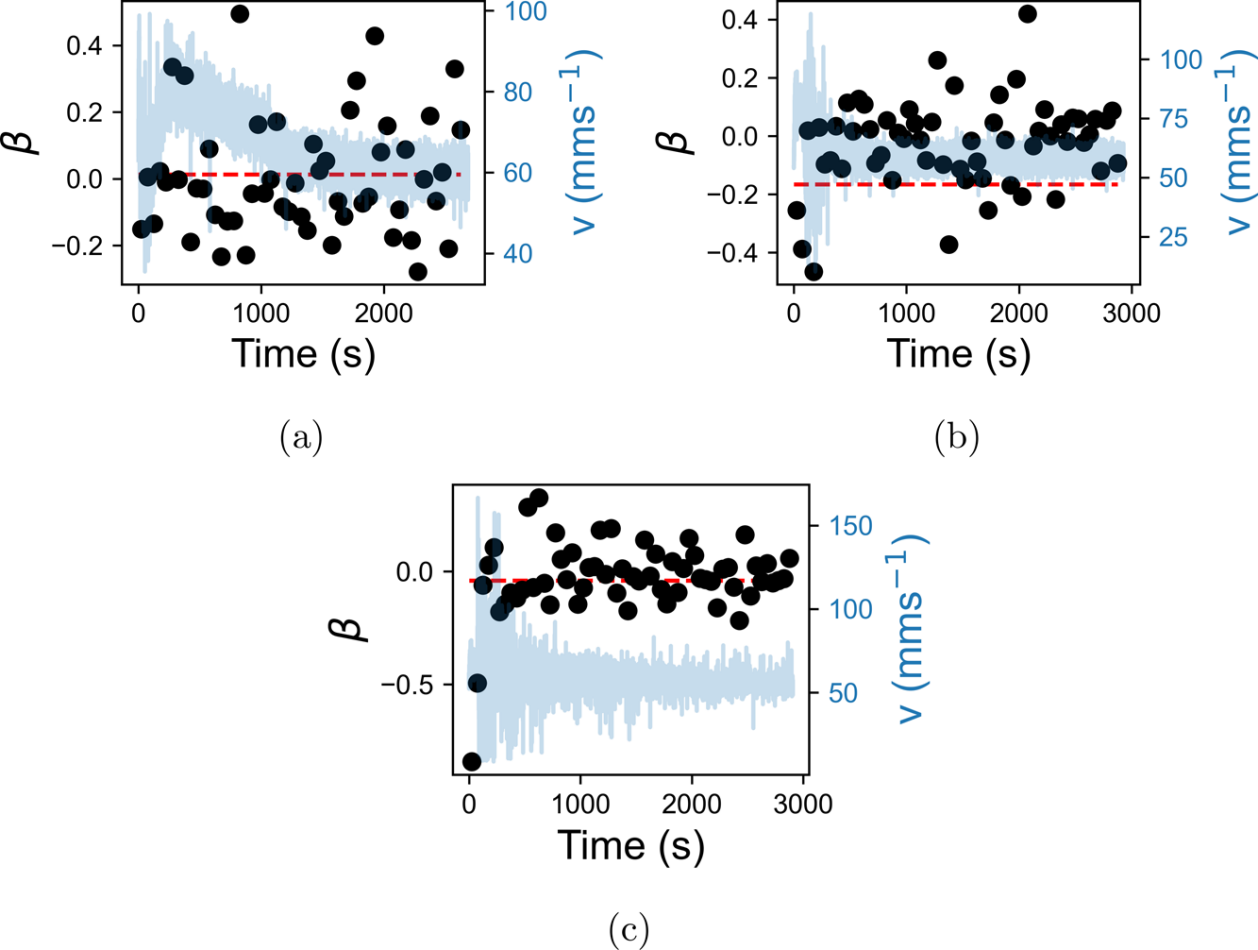
Exponent of the PSD of the velocity fluctuations of LGNs, β, in 50 s intervals after the addition of a salt solution. Increases to 0.5, 1.25, and 2% are in (a), (b), and (c), respectively. The preaddition value of β is shown on each plot as a red dotted line, and the velocity is plotted in the background.

It is common practice in industry to dynamically alter the formulation of products in situ. In order to study the effects of this on flow and to observe the mixing process, a salt solution was added to the LGN in the vessel while the pipe was in operation at 60 rpm to sequentially increase the NaCl concentration to 0.5, 1.25, and 2%. Transient velocity measurements were made over the course of 1 h, spanning before and after the salt solution was added. Figure 11 shows how the standard deviation, σ, of the velocity fluctuations varied in 50 s intervals after the addition of the salt solution, while Figure 12 shows how the exponent of the PSD, β, varies over the same time intervals. Figure 11 shows that the addition of salt solution is accompanied by an initial increase in σ, followed by a decrease to a new baseline when mixing is completed. The time taken to reach the baseline, *T*_*p*_, was calculated by finding the point at which the mean over five consecutive points of the percentage change in σ dropped below 5%. This resulted in *T*_*p*_ = 550 ± 25, 350 ± 25, and 1450 ± 25 s for the changes to 0.5, 1.25, and 2%, respectively. The baseline value of σ increases slightly after the increase to 0.5% NaCl but remains approximately constant after that, showing similarity to the results in Figure 7b. The time-dependent behavior of σ is not mirrored by β in Figure 12. Apart from a slight dip in the first few data points of Figure 12b,c depicting the increase to 1.25 and 2% NaCl, respectively, β remains close to zero at all times. Mixing therefore seems to have very little impact on the stability of the flow.

## Conclusions

OCT velocimetry was used to monitor the laminar pipe flow of lamellar gel networks (LGNs) of varying NaCl concentrations in an industrial-like setting for the first time. Fits of the velocity profile for power law plug flow to time-averaged velocity profiles produced estimated flow rates that matched well with those measured by an in-line Coriolis flow meter. The power law parameters also matched well with those previously found in a rheometer, despite different flow geometries and a factor of ∼10^4^ in volume. The partial fill of the pipe, biofouling, and the increased impact of the pulsatile nature of the flow due to reduced viscosity in the experiments with milk produced unsteady flow behavior. A smooth laminar flow profile was produced, but fits of the expected Hagen–Poiseuille flow did not produce a flow rate that exactly matched with that of the in-line flow meter, overshooting somewhat. Analysis of the transient behavior of the velocity fluctuations revealed stable, laminar flow at all measured pump speeds for the LGNs. In contrast, the milk was only laminar at the lowest speed, quickly transitioning to turbulence and showing power law energy scaling indicative of both intermittency and fully developed turbulence, as defined by Kolmogorov’s 5/3 law.^28^ The response of all fluids to the pulsatile pump could clearly be seen in their velocity oscillations and peaks in the fluctuation spectra. As pump speeds increased, the statistics of the fluctuations in all formulations of the LGN were found to become more Gaussian, as the fluid’s thixotropic nature played less of a role in dynamics. No such behavior was seen in the milk samples, as turbulent statistics began to dominate at higher rates. Though dynamic changes to the formulation of the LGNs were found to have very little effect on flow stability, the standard deviation of the velocity fluctuations temporarily increased, serving as an indicator of when the mixing process had been completed.

In-line OCT velocimetry can be used to monitor flow at higher rates than current industry standard measurement equipment while producing accurate estimates of the physical properties of complex fluids without the need for off-line analysis. Incorporated into industrial sites, it could be used in control loops to keep the flow steady, monitor the performance of the pump, determine the status of mixing, and monitor the physical properties of fluids, whether looking at perfecting formulations or detecting unwanted changes, potentially saving time and money.

## References

[ref1] Huang D.; Swanson E. A.; Lin C. P.; Schuman J. S.; Stinson W. G.; Chang W.; Hee M. R.; Flotire T.; Gregory K.; Puliafito C. A.; Fujimoto J. G. Optical Coherence Tomography. Science 1991, 254, 1178–1181. 10.1126/science.1957169. 1957169 PMC4638169

[ref2] Wang X. J.; Milner T. E.; Nelson J. S. Characterization of fluid flow velocity by optical Doppler tomography. Opt. Lett. 1995, 20, 1337–1339. 10.1364/OL.20.001337. 19859518

[ref3] Chen Z.; Milner T. E.; Dave D.; Nelson J. S. Optical Doppler tomographic imaging of fluid flow velocity in highly scattering media. Opt. Lett. 1997, 22, 64–66. 10.1364/OL.22.000064. 18183104

[ref4] Malm A.; Waigh T. A.; Jaradat S.; Tomlin R. Optical Coherence Tomography Velocimetry with complex fluids. Journal of Physics: Conference Series. 2015, 602, 012039 10.1088/1742-6596/602/1/012039.

[ref5] Manneville S. Recent experimental probes of shear banding. Rheol. Acta 2008, 47, 301–318. 10.1007/s00397-007-0246-z.

[ref6] Harvey M.; Waigh T. A. Optical coherence tomography velocimetry in controlled shear flow. Phys. Rev. E Stat Nonlin Soft Matter Phys. 2011, 83, 031502 10.1103/PhysRevE.83.031502. 21517502

[ref7] Jaradat S.; Harvey M.; Waigh T. A. Shear-banding in polyacrylamide solutions revealed via optical coherence tomography velocimetry. Soft Matter 2012, 8, 11677–11686. 10.1039/c2sm26395e.

[ref8] Malm A. V.; Harrison A. W.; Waigh T. A. Optical coherence tomography velocimetry of colloidal suspensions. Soft Matter 2014, 10, 8210–8215. 10.1039/C4SM01111B. 25181574

[ref9] Malm A. V.; Waigh T. A. Elastic turbulence in entangled semi-dilute DNA solutions measured with optical coherence tomography velocimetry. Sci. Rep. 2017, 7, 1186 10.1038/s41598-017-01303-4. 28442789 PMC5430809

[ref10] Watts Moore O.; Waigh T. A.; Mendoza C.; Kowalski A. Characterizing the rheology of lamellar gel networks with optical coherence tomography velocimetry. J. Rheol. 2023, 67, 589–600. 10.1122/8.0000599.

[ref11] Daneshi M.; Pourzahedi A.; Martinez D. M.; Grecov D. Characterising wall-slip behaviour of Carbopol gels in a fully-developed Poiseuille flow. J. Non-Newtonian Fluid Mech. 2019, 269, 65–72. 10.1016/j.jnnfm.2019.06.003.

[ref12] Malm A. V. OCT Velocimetry and X-ray Scattering Rheology of Complex Fluids; Ph.D. thesis; The University of Manchester, 2015.

[ref13] Guyon E.; Hulin J.-P.; Petit L.; Mitescu C. Physical Hydrodynamics, 2nd ed.; Oxford University Press, 2015; pp 90–137.

[ref14] De Schryver R.; De Schutter G. Insights in thixotropic concrete pumping by a Poiseuille flow extension. Appl. Rheol. 2020, 30, 77–101. 10.1515/arh-2020-0103.

[ref15] Datta A.; Tanmay V. S.; Tan G. X.; Reynolds G. W.; Jamadagni S. N.; Larson R. G. Characterizing the rheology, slip, and velocity profiles of lamellar gel networks. J. Rheol. 2020, 64, 851–862. 10.1122/8.0000011.

[ref16] Wang F.; Larson R. G. Thixotropic constitutive modeling of shear banding by boundary-induced modulus gradient in lamellar gel networks. J. Rheol. 2023, 67, 35–51. 10.1122/8.0000482.

[ref17] Avila M.; Barkley D.; Hof B. Transition to Turbulence in Pipe Flow. Annu. Rev. Fluid Mech. 2023, 55, 575–602. 10.1146/annurev-fluid-120720-025957.

[ref18] Mukund V.; Hof B. The critical point of the transition to turbulence in pipe flow. J. Fluid Mech. 2018, 839, 76–94. 10.1017/jfm.2017.923.

[ref19] Cotrell D. L.; McFadden G. B.; Alder B. J. Instability in pipe flow. Proc. Natl. Acad. Sci. U. S. A. 2008, 105, 428–430. 10.1073/pnas.0709172104. 18178623 PMC2206552

[ref20] Avila K.; Moxey D.; De Lozar A.; Avila M.; Barkley D.; Hof B. The onset of turbulence in pipe flow. Science 2011, 333, 192–196. 10.1126/science.1203223. 21737736

[ref21] Groisman A.; Steinberg V. Elastic turbulence in a polymer solution flow. Nature 2000, 405, 53–55. 10.1038/35011019. 10811214

[ref22] Samanta D.; Dubief Y.; Holzner M.; Schäfer C.; Morozov A. N.; Wagner C.; Hof B. Elasto-inertial turbulence. Proc. Natl. Acad. Sci. U.S.A. 2013, 110, 10557–10562. 10.1073/pnas.1219666110. 23757498 PMC3696777

[ref23] Agrawal N.; Choueiri G. H.; Hof B. Transition to Turbulence in Particle Laden Flows. Phys. Rev. Lett. 2019, 122, 114502 10.1103/PhysRevLett.122.114502. 30951357

[ref24] Hershey D.; Im C. S. Critical Reynolds number for sinusoidal flow of water in rigid tubes. AIChE J. 1968, 14, 807–809. 10.1002/aic.690140522.

[ref25] Falsetti H. L.; Carroll R. J.; Swope R. D.; Chen C. J. Turbulent blood flow in the ascending aorta of dogs. Cardiovasc. Res. 1983, 17, 427–436. 10.1093/cvr/17.7.427. 6883418

[ref26] Stettler J. C.; Hussain A. K. M. F. On transition of the pulsatile pipe flow. J. Fluid Mech. 1986, 170, 169–197. 10.1017/S0022112086000848.

[ref27] Trip R.; Kuik D. J.; Westerweel J.; Poelma C. An experimental study of transitional pulsatile pipe flow. Phys. Fluids 2012, 24, 014103 10.1063/1.3673611.

[ref28] Frisch U. Turbulence: The legacy of A.N. Kolmogorov, 1st ed.; Cambridge University Press: Cambridge, 1995.

[ref29] Taylor G. The Spectrum of Turbulence. Proc. R. Soc. London, Ser. A 1938, 164, 476 10.1098/rspa.1938.0032.

[ref30] Fercher A. F.; Drexler W.; Hitzenberger C. K.; Lasser T. Optical coherence tomography - principles and applications. Rep. Prog. Phys. 2003, 66, 239–303. 10.1088/0034-4885/66/2/204.

[ref31] Casugbo C.; Flanagan M.; Hough J. A.; Naughton J. M.; Serridge D. Composition; European Patent Office, 2014,.

[ref32] Cunningham G. E.; Alberini F.; Simmons M. J.; O’Sullivan J. J. Understanding the effects of processing conditions on the formation of lamellar gel networks using a rheological approach. Chem. Eng. Sci. 2021, 242, 116752 10.1016/j.ces.2021.116752.

[ref33] Iwata T. In Cosmetic Science and Technology: Theoretical Principles and Applications, Sakamoto K.; Lochhead R.; Maibach H.; Yamashita Y., Eds.; Elsevier Inc., 2017; Chapter 25, pp 415–447.

[ref34] Junginger H. Colloidal structures of O/W creams. Pharmaceutisch Weekblad Scientific Edition 1984, 6, 141–149. 10.1007/BF01954041. 6483570

[ref35] Eccleston G. M. Functions of mixed emulsifiers and emulsifying waxes in dermatological lotions and creams. Colloids Surf., A 1997, 123–124, 169–182. 10.1016/S0927-7757(96)03846-0.

[ref36] Warriner H. E.; Idziak S. H.; Slack N. L.; Davidson P.; Safinya C. R. Lamellar biogels: Fluid-membrane-based hydrogels containing polymer lipids. Science 1996, 271, 969–973. 10.1126/science.271.5251.969. 8584932

[ref37] Salmon J. B.; Manneville S.; Colin A. Shear banding in a lyotropic lamellar phase. I. Time-averaged velocity profiles. Physical Review E - Statistical Physics, Plasmas, Fluids, and Related Interdisciplinary Topics 2003, 68, 051503 10.1103/PhysRevE.68.051503. 14682801

[ref38] Roux D.; Nallet F.; Diat O. Rheology of Lyotropic Lamellar Phases. Europhys. Lett. 1993, 24, 53–58. 10.1209/0295-5075/24/1/009.

[ref39] Diat O.; Roux D.; Nallet F. Effect of shear on a lyotropic lamellar phase. Journal de Physique II, EDP Sciences 1993, 3, 1427–1452. 10.1051/jp2:1993211.

[ref40] Hoffmann H.; Ulbricht W. Surfactant gels. Curr. Opin. Colloid Interface Sci. 1996, 1, 726–739. 10.1016/S1359-0294(96)80074-4.

[ref41] Medronho B.; Olsson U.; Schmidt C.; Galvosas P. Transient and steady-state shear banding in a lamellar phase as studied by rheo-NMR. Zeitschrift fur Physikalische Chemie 2012, 226, 1293–1313. 10.1524/zpch.2012.0313.

[ref42] Wilkins G. M.; Olmsted P. D. Vorticity banding during the lamellar-to-onion transition in a lyotropic surfactant solution in shear flow. Eur. Phys. J. E 2006, 21, 133–143. 10.1140/epje/i2006-10053-9. 17139454

[ref43] Manneville S.; Salmon J. B.; Colin A. A spatio-temporal study of rheo-oscillations in a sheared lamellar phase using ultrasound. Eur. Phys. J. E 2004, 13, 197–212. 10.1140/epje/e2004-00046-y. 15052429

[ref44] Sollich P.; Lequeux F.; Hébraud P.; Cates M. E. Rheology of soft glassy materials. Phys. Rev. Lett. 1997, 78, 2020–2023. 10.1103/PhysRevLett.78.2020.

[ref45] Guo J.; Meroney R. N. Theoretical solution for laminar flow in partially-filled pipes. Journal of Hydraulic Research 2013, 51, 408–416. 10.1080/00221686.2013.784881.

[ref46] Ng H. C.-H.; Cregan H. L. F.; Dodds J. M.; Poole R. J.; Dennis D. J. C. Partially filled pipes: experiments in laminar and turbulent flow. J. Fluid Mech. 2018, 848, 467–507. 10.1017/jfm.2018.345.

[ref47] Womersley J. R. Method for the calculation of velocity, rate of flow and viscous drag in arteries when the pressure gradient is known. Journal of Physiology 1955, 127, 553–563. 10.1113/jphysiol.1955.sp005276. 14368548 PMC1365740

[ref48] Uchida S. The pulsating viscous flow superposed on the steady laminar motion of incompressible fluid in a circular pipe. Zeitschrift f ü r angewandte Mathematik und Physik ZAMP 1956, 7, 403–422. 10.1007/BF01606327.

[ref49] Shemer L.; Wygnanski I.; Kit E. Pulsating flow in a pipe. J. Fluid Mech. 1985, 153, 313–337. 10.1017/S0022112085001276.

[ref50] Denison E. B.; Stevenson W. H.; Fox R. W. Pulsating laminar flow measurements with a directionally sensitive laser velocimeter. AIChE J. 1971, 17, 781–787. 10.1002/aic.690170405.

[ref51] Ting K.; Liu Y.-F.; Tian-Li G.; Lu-Hua Z. Relationships between viscosity and the contents of macromolecular substances from milk with different storage styles. Food Science and Technology 2016, 4, 49–56. 10.13189/fst.2016.040401.

[ref52] Steinberg V. Scaling Relations in Elastic Turbulence. Phys. Rev. Lett. 2019, 123, 234501 10.1103/PhysRevLett.123.234501. 31868477

[ref53] Hogendoorn W.; Chandra B.; Poelma C. Onset of turbulence in particle-laden pipe flows. Phys. Rev. Fluids 2022, 7, L042301 10.1103/PhysRevFluids.7.L042301.

